# Preparing a Cavity for Porcelain Inlays

**Published:** 1904-05

**Authors:** N. S. Jenkins

**Affiliations:** DRESDEN, GER.


### PREPARING A CAVITY FOR PORCELAIN INLAYS.

N. S. JENKINS, DRESDEN, GER.

It is indispensable that the cavity should be shaped so that the matrix can be perfectly made and withdrawn without changing the shape in ever so slight a degree. To this we need not hesitate to sacrifice frail walls, especially upon occlusal surfaces, as the crowns of molars and bicuspids and the palatal walls of upper incisors. In all approximal cavities space must be gained by wedging or filing. At all occlusal surfaces the walls of the cavity must be at right angles, and thin edges should be avoided as far as possible. All the walls of the cavity should be as nearly at right angles as circumstances permit. Occasionally some decayed dentin can be allowed to remain until the completed filling is ready to be set, if its premature removal would necessitate undercuts, but usually every particle of decay can be removed with advantage in the first preparation of the cavity. All the edges should be clearly defined and well-polished, that it may be possible to burnish the edges without injury to the matrix. It is well to polish also the interior of the cavity, as a smooth surface facilitates the removal of the matrix.—*International.*

